# Communication and Contextual Factors in Robotic-Assisted Surgical Teams: Protocol for Developing a Taxonomy

**DOI:** 10.2196/54910

**Published:** 2024-06-17

**Authors:** Kyi Phyu Nyein, Claire Condron

**Affiliations:** 1 SIM Centre for Simulation Education and Research Royal College of Surgeons in Ireland University of Medicine and Health Sciences Dublin Ireland

**Keywords:** communication, teams, robotic surgery, robotic-assisted, simulation

## Abstract

**Background:**

Robotic-assisted surgery (RAS) has been rapidly integrated into surgical practice in the past few decades. The setup in the operating theater for RAS differs from that for open or laparoscopic surgery such that the operating surgeon sits at a console separate from the rest of the surgical team and the patient. Communication and team dynamics are altered due to this physical separation and visual barriers imposed by the robotic equipment. However, the factors that might comprise effective communication among members of RAS teams and the contextual factors that facilitate or inhibit effective communication in RAS remain unknown.

**Objective:**

We aim to develop a taxonomy of communication behaviors and contextual factors that influence communication in RAS teams. We also aim to examine the patterns of communication behaviors based on gender.

**Methods:**

We will first perform a scoping review on communication in RAS to develop a preliminary taxonomy of communication based on the existing literature. We will then conduct semistructured interviews with RAS team members, including the surgeon, assisting surgeon or trainee, bedside or first assistant, nurses, and anesthetists. Participants will represent different disciplines, including urology, general surgery, and gynecology, and have a range of experiences in RAS. We will use a reflexive thematic analysis to analyze the data and further refine the taxonomy. We will also observe live robotic surgeries at Royal College of Surgeons in Ireland (RCSI)–affiliated hospitals. We will observe varying lengths and conditions of RAS procedures to a capture a wide range of communication behaviors and contextual factors to help finalize the taxonomy. Although we anticipate conducting 30 interviews and 30 observations, we will collect data until we achieve data sufficiency. We will conduct data collection in parallel with data analysis such that if we identify a new behavior in an interview, we will follow up with questions related to that behavior in additional interviews and/or observations.

**Results:**

The taxonomy from this project will include a list of actionable communication behaviors, contextual factors, their descriptions, and examples. As of May 2024, this project has been approved by the RCSI Research and Ethics Committee. Data collection started in June 2024 and will continue throughout the year. We plan to publish the findings as meaningful results emerge in our data analysis in 2024 and 2025.

**Conclusions:**

The results from this project will be used to observe and train surgical teams in a simulated environment to effectively communicate with each other and prevent communication breakdowns. The developed taxonomy will also add to the knowledge base on the role of gender in communication in RAS and produce recommendations that can be incorporated into training. Overall, this project will contribute to the improvement of communication skills of surgical teams and the quality and safety of patient care.

**International Registered Report Identifier (IRRID):**

PRR1-10.2196/54910

## Introduction

There has been exponential growth in establishing and integrating robotic-assisted surgery (RAS) into surgical practice and training in the last few decades. For example, the use of RAS for all general surgery procedures increased from ~2% to ~15% from 2012 to 2018 [1]. Compared with open or laparoscopic surgery, RAS reduces postoperative pain, promotes faster recovery time, and provides better patient outcomes [2]. However, the introduction of a robotic system into the operating theater changes the spatial configuration of the surgical team and patient. In RAS, the surgeon sits at a console and is physically separated from the surgical team and patient who remain in the sterile field. As the surgeon places their head inside the console to look at the video feed of the surgical site, they no longer have a direct view of physical movements and nonverbal cues from the surgical team. The surgical team also faces the robotic operating equipment, which obstructs their views of each other. Consequently, the surgeon and the rest of the surgical team are dependent on explicit, descriptive communication not only to give and respond to instructions and requests but also to provide updates on the status of the patient and robotic system. Thus, RAS entirely changes communication and team dynamics in the surgical team, raising the potential for miscommunication and misunderstandings that may threaten the potential for enhanced outcomes.

Nontechnical skills such as communication are critical for improving surgeons’ performance, operative workflow, and patient outcomes and for reducing adverse events [3,4]. Although communication has been identified as one of the essential nontechnical skills in robotic surgery [3,5], little is known about how the surgical teams communicate most effectively in RAS. This limited understanding about what constitutes effective communication in RAS is problematic because failed communication is the second most common factor contributing to surgical errors [6]. Thus, this protocol describes a project that aims to develop a taxonomy of communication behaviors and contextual factors that facilitate or inhibit effective communication in RAS.

Previous studies have developed assessment or rating tools for nontechnical skills in RAS using behavioral marker methodology [3,5]. Schreyer et al [4] identified leadership and management, teamwork and cooperation, problem-solving and decision-making, and situational awareness as essential nontechnical skills. However, their rating tool did not focus specifically on communication but rather on nontechnical skills as a whole. In contrast, Manuguerra et al [3] and Raison et al [5] identified communication as an essential nontechnical skill. However, their behavioral markers were not actionable from the perspective of training, including “effective verbal communication whilst at the console,” “appropriate interaction with beside assistant surgeon,” and “presence of feedback” [3,5]. As an example, team members cannot coordinate with each other if they do not effectively communicate their needs. Therefore, this project goes beyond the current state of this field and further examines communication specifically as a key nontechnical skill in RAS as it is fundamental to other nontechnical skills.

Moreover, Manuguerra et al [3] and Raison et al [5] developed rating tools only for surgeons. However, RAS changes existing roles not only for surgeons but also for other surgical team members. For example, nurses in RAS are expected to have more technical knowledge and coordination than required in traditional surgery [7]. They are also expected to speak up and share information pertaining to issues occurring outside of the surgeon’s field of vision that might help improve efficiency in the operating theater or patient safety [8]. As a result, there are changes in role-based communication needs and expectations that must be considered. Therefore, this project goes beyond previous studies that examined nontechnical skills only for surgeons by further examining communication among all RAS team members.

Gender also influences the perceptions, experiences, teamwork, and performance of health care professionals in the operating theater, thereby affecting patient care and outcomes. For example, compared to men, women are generally less listened to when they speak up and share concerns about the patient in the operating theater [9]. In addition, male and female health care professionals engage in their clinical practices differently, which leads to different patient outcomes. Examples of this difference include evidence that female physicians are more likely to follow clinical guidelines and engage in more patient-centered communication compared to male physicians, and patients treated by female physicians experience lower mortality and readmission rates than those treated by male physicians [10]. Thus, it is important to understand how gender influences surgical teams’ training, learning, teamwork, and performance in RAS. However, the role of gender in robotic surgery has not been studied in previous research. As there are changes in team roles and dynamics in robotic surgery, this project addresses this research gap and examines the role of gender in communication among robotic surgical teams.

This project has two important contributions to RAS. First, the taxonomy developed from this project will include a list of actionable communication behaviors along with contextual factors and their associated descriptions and examples. We anticipate that the taxonomy will be used for behavioral observations and training surgical team members to effectively communicate with each other and prevent communication failures. Second, this project will answer important questions related to the gender dimension (eg, male vs female surgical team members engaging in a specific communication behavior) that can be taken into consideration in their training.

## Methods

### Scoping Review

#### Literature Search

We will first perform a scoping review to examine the existing literature on communication in RAS and identify the gaps in the literature according to the methodological guidance of Peters et al [11]. The review will be reported based on the PRISMA-ScR (Preferred Reporting Items for Systematic reviews and Meta-Analyses extension for Scoping Reviews) guideline and checklist [12].

We will search the following databases: PsycINFO, CINAHL, PubMed, Cochrane, Embase, MEDLINE, and Web of Science. We will also search the grey literature in the Scopus and Google Scholar databases. The search terms and strategy are outlined in Textbox 1.

**Search strategy for the scoping review.**

1: Title/abstract

- robotic surg* OR “robotic surgery” OR “robot-assisted surgery” OR “robot assisted surgery” OR “robotic assisted surgery” OR “robotic-assisted surgery” OR robotic surgical procedure* OR robotic surg* team* OR “minimally invasive surgery”

2: Medical Subject Heading (MeSH) terms or subject terms

- robotic surgery

3: 1 OR 2

4: Title/abstract

- “non-technical skills” OR “non technical skills” OR “nontechnical skills” OR communicat* OR coordinat* OR cooperat* OR collaborat* OR teamwork OR team process* OR interpersonal skill* OR “information sharing” OR non-technical skill* OR team dynamic*

5: MeSH terms or subject terms

- communication

6: 4 OR 5

7: 3 AND 6 with the following syntax:

(“robotic surg*”[Title/Abstract] OR “robotic surgery”[Title/Abstract] OR “robot-assisted surgery”[Title/Abstract] OR “robot-assisted surgery”[Title/Abstract] OR “robotic-assisted surgery”[Title/Abstract] OR “robotic-assisted surgery”[Title/Abstract] OR “robotic surgical procedure*”[Title/Abstract] OR (((“robot”[All Fields] OR “robot s”[All Fields] OR “robotically”[All Fields] OR “robotics”[MeSH Terms] OR “robotics”[All Fields] OR “robotic”[All Fields] OR “robotization”[All Fields] OR “robotized”[All Fields] OR “robots”[All Fields]) AND “surg*”[All Fields]) AND “team*”[Title/Abstract]) OR “minimally invasive surgery”[Title/Abstract] OR “robotic surgical procedures”[MeSH Terms]) AND (“non-technical skills”[Title/Abstract] OR “non-technical skills”[Title/Abstract] OR “nontechnical skills”[Title/Abstract] OR “communicat*”[Title/Abstract] OR “coordinat*”[Title/Abstract] OR “cooperat*”[Title/Abstract] OR “collaborat*”[Title/Abstract] OR “teamwork”[Title/Abstract] OR “team process*”[Title/Abstract] OR “interpersonal skill*”[Title/Abstract] OR “information sharing”[Title/Abstract] OR “non technical skill*”[Title/Abstract] OR “team dynamic*”[Title/Abstract] OR “communication”[MeSH Terms])

#### Study Selection and Data Extraction

We will select studies for the review based on the inclusion and exclusion criteria outlined in Table 1. We will then extract information relevant to our research focus (ie, communication in RAS) using a charting table. We will analyze the data using descriptive statistics (eg, frequency). Finally, we will report the results according to PRISMA-ScR guidelines as well as in diagrams and/or tables as appropriate. This scoping review will result in the preliminary taxonomy of communication and contextual factors in RAS teams.

**Table 1:** Inclusion and exclusion criteria in the scoping review.

Category	Inclusion criteria	Exclusion criteria
Publication date	Between January 2010 and December 2023	Before 2010
Population	Health care professionals	Medical students
Context	Simulation, hospitals, medical centers; teaching/learning environment; effects of environmental, external, or contextual factors (eg, stress, audio and video issues) on communication	Community
Type of surgery	Robotic-assisted surgery or comparison of robotic surgery to other types of surgery	Laparoscopic, open
Sources	Peer-reviewed articles, conference proceedings, theses, and dissertations	Books, periodicals, magazines, policy documents, and websites
Language	English	Non-English
Study design	Quantitative, qualitative, and mixed methods; training, assessment, rating tool, checklist, protocol	All types of reviews

### Interviews

This project will take place at the Royal College of Surgeons in Ireland (RCSI). We will use purposive and convenience sampling to recruit participants. We will invite participants through the RCSI’s surgical networks. We will conduct one-on-one semistructured interviews with members of the surgical teams, including the consultant/attending surgeon, assisting surgeon or trainee, bedside or first assistant, nurses, and anesthetists. Participants will represent different disciplines, including urology, general surgery, and gynecology, and have a range of experiences in robotic surgery. The interviews will take place in person or virtually and will be audio-recorded and transcribed. A sample interview questions is “In robotic-assisted surgery, what does it look like when communication is effective in the team?”

Although we anticipate conducting 30 interviews, we will collect data until we achieve data sufficiency (ie, sufficient data to answer the research questions capturing both the uniqueness of the communication experience and its socially constructed meaning) [13] and an equal representation of male and female voices. In qualitative research, sample size depends on several factors such as study purpose, sample specificity, and quality of exchange between researchers and participants. For example, if participants offer rich accounts of their experiences, the sample size required may be lower than otherwise. By contrast, a study with a broad aim and limited theoretical background requires a higher sample size.

We will analyze the data in parallel to data collection. We will use reflexive thematic analysis, an iterative process where researchers interpret and analyze patterns of behaviors while being aware of their own assumptions, experiences, and social positions (eg, with regard to gender) [14]. We will identify and develop themes of communication and contextual factors. We will also calculate interrater agreement with an adequate proportion of the data. If we find a new theme, we will clarify or ask further questions in the following interviews. Through this iterative process, we will further refine the taxonomy.

### Observations

We will conduct interviews and observations in parallel to allow these findings to inform each other as the period of data collection and iterative data analysis proceeds. We will observe live robotic surgical procedures at hospitals affiliated with the RCSI. The RCSI Group is comprised of several hospitals, including Beaumont Hospital, which provides a national and regional service to Dublin and the eastern and northern regions of Ireland. We will invite participants through the RCSI’s surgical networks. A researcher will be present in the operating theater and take notes at an optimal distance to hear any communication occurring among the surgeon, assisting surgeon, first assistant, nurses, and anesthetist. Excel spreadsheets will be used to record the frequency of the behaviors observed. Moreover, we will observe varying lengths of robotic surgical procedures to capture a wide range of communication behaviors, as certain parts of a procedure are more challenging and create conditions that require different strategies of management and communication. Although we anticipate observing 30 robotic surgical procedures, we will collect data until we achieve data sufficiency.

We will analyze the data and further refine the taxonomy. We will also calculate interrater agreement with an adequate proportion of observations. If we find a new behavior or event during an observation, we will follow up with additional interviews and/or observations. We will pursue this iterative process until we achieve data sufficiency. The final outcome of the iterative process is a valid and reliable taxonomy of communication behaviors and contextual factors, their descriptions, and examples that can be used for behavioral observations.

### Ethical Considerations

The study was approved by the RCSI Research and Ethics Committee in May 2024 (reference number: REC202309006). We will seek informed consent from the surgical team members and patients, and they can withdraw from the study at any point without repercussions. Participants will not receive any compensation. Data will be pseudonymized and stored on the secure, encrypted RCSI OneDrive that only the research team has access to. The data sets, including raw data, will not be openly available as they contain personal information of participants (eg, gender) and their personal experiences that are confidential.

### Dissemination and Secondary Use of Data

The taxonomy resulting from this project will serve as the metadata that will include codes used in data analysis, their description, and examples. Researchers can use the taxonomy to replicate the study or for future research. The taxonomy will be published in open access journals and/or made publicly available via appropriate repositories (eg, Zenodo, RCSI Repository, Open Research Europe). Request for access to the data may be considered if individuals have received training on research ethics and General Data Protection Regulation and approvals from appropriate research ethics committees.

## Results

The data collected will include interviews (audio files, transcripts, and a codebook) and observational notes (Excel sheet). The taxonomy will include a list of communication behaviors and contextual factors, their descriptions, and examples. This project has been funded by a Marie Skłodowska-Curie Actions Fellowship (grant 101107170) of the European Union from September 2023 to September 2025. As of June 2024, we have screened abstracts and titles of 5024 articles and the full texts of 182 articles. We are preparing to assess 79 included articles. Moreover, we have conducted one interview and will continue data collection and analysis throughout the year. We aim to publish the findings as meaningful results emerge throughout our data analysis in 2024 and 2025.

## Discussion

### Anticipated Results

RAS differs from open or laparoscopic surgery mainly because of the surgeon’s separation from the rest of the surgical team and the patient and the presence of the robotic operating equipment in the operating theater. This setup inherently changes communication and team dynamics in RAS teams. This protocol describes a project that aims to develop a taxonomy of communication and contextual factors that facilitate or inhibit effective communication in RAS. The taxonomy will include a list of communication behaviors and contextual factors in RAS, their descriptions, and examples. We also anticipate obtaining results related to the gender dimension, such as whether and/or how male and female surgeons engage in similar or different patterns of communication behaviors.

### Comparison With Prior Work

Compared with previous studies that examined mainly nontechnical skills in RAS [3-5], this project focuses specifically on communication and its contextual factors that facilitate or inhibit effective communication in RAS teams. As communication is fundamental to other nontechnical skills, the taxonomy of communication developed from this project can be used for behavioral observations and training focusing specifically on communication. In addition, previous studies have used behavioral marker methodology to develop rating tools for nontechnical skills in RAS. Behavioral marker methodology categorizes various behaviors into broad behavioral classes [15]. Although this approach provides an overall assessment of nontechnical skills, some behaviors in a given behavioral class may not occur frequently and this method is susceptible to observer bias [15]. For example, one behavior might stand out to an observer who then rates the entire behavioral class favorably. Alternatively, the taxonomy from this project will focus on specific and defined communication behaviors that are easier to observe and train and less susceptible to observer bias. Thus, this project has unique contributions to improving communication skills in RAS teams.

### Limitations

We acknowledge possible limitations in this project. We are collecting data in the da Vinci robotic surgical system (Intuitive Surgical), as it is the platform most surgeons are currently familiar with. However, there are other robotic systems such as the Hugo RAS System (Medtronic) and Versius (CMR Surgical). There might be differences in communication according to different system setups. Moreover, we will only examine core communication behaviors that can be trained across different disciplines or specialties. There might be discipline-specific communication as well as contexts (eg, emergency situation) that need to be incorporated into training. Therefore, future research should further examine RAS team communication in different contexts.

### Conclusions

The results from this project will serve as training materials to observe and train surgical teams in a simulated environment to effectively communicate with each other and prevent communication failures. Simulation provides a safe environment that mimics a real hospital for learning and practicing surgical skills and techniques. Thus, the results will be applied in simulation training focusing on communication for surgical teams. We will also answer important questions related to the gender dimension. The results will inform behaviors and contexts that need to be emphasized in simulation training and address gender gaps. In conclusion, this project aims to improve the communication skills of RAS teams so that they are competent and responsible in effectively communicating with each other and using robotics to deliver safe patient care.

## Appendix

Multimedia Appendix 1 Peer-review report by the European Commission - Horizon Europe Framework Programme - Royal College of Surgeons in Ireland.

## Abbreviations

PRISMA-ScR: Preferred Reporting Items for Systematic reviews and Meta-Analyses extension for Scoping Reviews
RAS: robotic-assisted surgery
RCSI: Royal College of Surgeons in Ireland
