# Supplementary material for: Communication and Contextual Factors in Robotic-Assisted Surgical Teams: Protocol for Developing a Taxonomy
Source: JMIR Res Protoc. 2024 Jun 17;13:e54910. doi: 10.2196/54910 (PMC11217702; doi:10.2196/54910)
Supplement: Multimedia Appendix 1 [file resprot_v13i1e54910_app1.pdf]

Proposal Evaluation Form

	**EUROPEAN COMMISSION**<br><br>Horizon Europe Framework Programme (HORIZON)	**Evaluation Summary Report - Postdoctoral Fellowships**

**Call:**	HORIZON-MSCA-2022-PF-01
**Type of action:**	HORIZON-TMA-MSCA-PF-EF
**Proposal number:**	101107170
**Proposal acronym:**	ROSE
**Duration (months):**	24
**Proposal title:**	Developing and Applying a Taxonomy of Communication Behaviours and Contextual Factors in Robotic Surgery
**Activity:**	EF-SOC

N.	Proposer name	Country	Total eligible costs	%	Grant Requested	%
1	ROYAL COLLEGE OF SURGEONS IN IRELAND	IE	0	-		
	Total:		0			

Abstract:

Robotic surgery is being rapidly and widely incorporated into surgical practice, with the current focus of training on equipment, mechanics, and technical skills. The operating theatre setup for robotic surgery is inherently different from the traditional arrangement, with the operating surgeon at a console that is remote from the rest of the surgical team and patient. The team faces significant challenges due to this physical separation and the visual barrier imposed by the robotic operating equipment. As a result, leadership and team communication are dynamically altered. Nonverbal communication and cues need to be replaced by explicit and descriptive communication. The overarching aim of my project is to examine what comprises effective communication by robotic surgical teams. I will observe verbal communication of surgical teams being trained in robotic surgical skills in a simulated operating theatre in the world-class RCSI SIM. I will also interview them about their experiences of effective and ineffective communication in performing robotic surgeries. I will develop a taxonomy of communication behaviours and contextual factors that facilitate or inhibit effective communication, their descriptions, and examples. Using the taxonomy, I will observe live robotic surgeries in RCSI hospitals and code the frequency, time, and sequence of communication behaviours and contextual factors using an innovative method called event coding. The taxonomy from my research will serve as training materials for simulation training focusing on communication for robotic surgical teams, and will contribute to the continuous improvement of modern surgery and the quality and safety of patient care. With the support of Dr Claire Condron and Prof Walter Eppich, this fellowship will help me pursue a successful career as an academic scientist or director of a simulation centre that makes meaningful impacts on the performance and well-being of healthcare professionals and patients.

Evaluation Summary Report

Evaluation Result

Total score: 96.80% (Threshold: 70/100.00)

Criterion 1 - Excellence

Score: **4.80** (Threshold: 0/5.00 , Weight: 50.00%)

Scanned with CamScanner

• Quality and pertinence of the project's research and [redacted]novation objectives (and the extent to which they are ambitious, and go beyond the state of the art).
• Soundness of the proposed methodology (including interdisciplinary approaches, consideration of the gender dimension and other diversity aspects if relevant for the research project, and the quality of open science practices).
• Quality of the supervision, training and of the two-way transfer of knowledge between the researcher and the host
• Quality and appropriateness of the researcher's professional experience, competences and skills.

STRENGHTS

• The objective of the proposal is clear (communication in robotic surgery).

• The proposed work is ambitious in addressing new challenges in the state-of-the-art in the field of robotic surgery and modern medicine.

• The research and innovation objectives are well-formulated and very convincing.

• The proposed methodology is sounding and the measures to tackle methodological challenges are well identified.

• The interdisciplinary aspects of the proposal are well-addressed, by pointing out the integration between more humanistic and more technological aspects in the design of the different studies.

• The gender aspects are well described, with details on the focus of the proposal on gender aspects that can influence behavior of health professionals.

• Open science practices are taken in good consideration, with indication of clear actions that the researcher would do to assure spread of the research findings.

• The (main) supervisor has an excellent track record and is totally qualified to successfully supervise a project like this one. The co-supervisor brings complementary expertise (e.g. qualitative research) that adds value and can contribute significantly to the success of the project.

• The proposal presents a detailed and convincing description of the training activities for the researcher, including both scientific/methodological skills and transferable skills relevant to the researcher's future research career.

• The host institution and the researcher would clearly benefit from the two-way transfer of knowledge, which is well-articulated through several specific activities, in a very convincing way.

• The researcher has good general research competences and skills, especially considering all previous research activities and involvement in projects. The publication level is fair for the career stage.

WEAKNESSES

• It is not sufficiently demonstrated the professional experience, competences and skills in psychology and team organization which the researcher role would demand in view of the complexity of the specific setting of medical surgery.

Criterion 2 - Impact

Score: **4.80** (Threshold: 0/5.00 , Weight: 30.00%)

Scanned with CamScanner

• Credibility of the measures to enhance the career per[redacted]ectives and employability of researchers and contribution to their skills development.
• Suitability and quality of the measures to maximise expected outcomes and impacts, as set out in the dissemination and exploitation plan, including communication activities.
• The magnitude and importance of the project's contribution to the expected scientific, societal and economic impacts.

STRENGHTS

• The contribution of the proposed measures to the development of the researcher's skills is convincing, since it would allow the development of skills that the researcher already has to some extent (e.g., qualitative analysis, transferable skills) and the acquisition of new ones (e.g., event coding).

• The proposed measures have a clear potential to substantially enhance the researcher's career perspectives and employability, particularly in terms of developing scientific and transferable skills and expanding the researcher's network of collaborators.

• The proposal identifies a series of specific dissemination and exploitation actions for the results of the project, which are carefully planned. For these, specific support resources are clearly described, and specific target groups are properly identified, adding value and credibility to the planning.

• The strategy for the management of the intellectual property is sound and includes all the relevant elements that may require consideration in this respect including potential patent protection.

• The proposal clearly describes how effective communication activities, via a variety of channels (e.g. social media, popular science events etc.) would be put into practice, which has a clear potential to ensure public engagement with the work conducted in the project.

• The proposal argues in a convincing way its high social and economic impact (e.g., in relation to the training of surgeons, reduction of errors during surgeries, and reduction of costs associated with those), which can clearly last beyond the duration of the project, given the growing trend towards the incorporation of robotic tools in surgery.

WEAKNESSES

• The scientific impact of the proposal is not fully elaborated. In addition, the potential impact on broader scientific understanding of communication in robotic surgery is not sufficiently clarified.

Criterion 3 - implementation

Score: **5.00** (Threshold: 0/5.00 , Weight: 20.00%)

• Quality and effectiveness of the work plan, assessment of risks and appropriateness of the effort assigned to work packages.
• Quality and capacity of the host institutions and participating organisations, including hosting arrangements.

STRENGHTS

• The workplan is well structured under five WPs that contain clear tasks, activities, milestones and deliverables.

• The Gantt chart is consistent and complete in relation to the whole work plan.

• The risk planned is well-developed, with a clear contingency plan to mitigate eventual difficulties that can impact the conduct of the proposal (e.g., in recruiting participants).

• The hosting arrangements are well justified, and they align perfectly with the needs of the project.

• The host institution clearly has the capacity, in terms of infrastructure, logistics, and facilities, to cover all the project's requirements. The host institution's extensive previous experience in projects of a similar nature contributes to the credibility of this aspect.

Scope of the application

Scanned with CamScanner

Status: **Yes**

Associated with document Ref. Ares(2023)465938 - 20/01/2023

Comments (in case the proposal is out of scope)
Not provided

Exceptional funding

A third country participant/international organisation not listed in the General Annex to the Main Work Programme may exceptionally receive funding if their participation is essential for carrying out the project (for instance due to outstanding expertise, access to unique know-how, access to research infrastructure, access to particular geographical environments, possibility to involve key partners in emerging markets, access to data, etc.). (For more information, see the HE programme guide)

Please list the concerned applicants and requested grant amount and explain the reasons why.

Based on the information provided, the following participants should receive exceptional funding:
Not provided

Based on the information provided, the following participants should NOT receive exceptional funding:
Not provided

Use of human embryonic stem cells (hESC)

Status: **No**

If YES, please state whether the use of hESC is, or is not, in your opinion, necessary to achieve the scientific objectives of the proposal and the reasons why. Alternatively, please state if it cannot be assessed whether the use of hESC is necessary or not, because of a lack of information.
Not provided

Use of human embryos

Status: **No**

If YES, please explain how the human embryos will be used in the project.
Not provided

Activities excluded from funding

Status: **No**

If YES, please explain.
Not provided

Do no significant harm principle

Status: **Not applicable**

If Partially/No/Cannot be assessed please explain
Not provided

Exclusive focus on civil applications

Status: **Yes**

Scanned with CamScanner

If NO, please explain.

Not provided

Artificial Intelligence

Status: **No**

If YES, the technical robustness of the proposed system must be evaluated under the appropriate criterion.

Overall comments

Not provided

Scanned with CamScanner

Digitally sealed by the European Commission
Date: 2023.01.20 20:22:05 CET

This electronic receipt is a digitally signed version of the document submitted by your organisation. Both the content of the document and a set of metadata have been digitally sealed.

This digital signature mechanism, using a public-private key pair mechanism, uniquely binds this eReceipt to the modules of the Funding & Tenders Portal of the European Commission, to the transaction for which it was generated and ensures its full integrity. Therefore a complete digitally signed trail of the transaction is available both for your organisation and for the issuer of the eReceipt.

Any attempt to modify the content will lead to a break of the integrity of the electronic signature, which can be verified at any time by clicking on the eReceipt validation symbol.

More info about eReceipts can be found in the FAQ page of the Funding & Tenders Portal.

(https://ec.europa.eu/info/funding-tenders/opportunities/portal/screen/support/faq)

Scanned with CamScanner
